# Optimal-Band Analysis for Chlorophyll Quantification in Rice Leaves Using a Custom Hyperspectral Imaging System

**DOI:** 10.3390/plants13020259

**Published:** 2024-01-16

**Authors:** Panuwat Pengphorm, Sukrit Thongrom, Chalongrat Daengngam, Saowapa Duangpan, Tajamul Hussain, Pawita Boonrat

**Affiliations:** 1 Division of Physical Science, Faculty of Science, Prince of Songkla University, Hat Yai 90110, Songkhla, Thailand; panuwat@narit.or.th (P.P.); sukrit@narit.or.th (S.T.); chalongrat.d@psu.ac.th (C.D.); 2 National Astronomical Research Institute of Thailand (Public Organization), Mae Rim 50180, Chiang Mai, Thailand; 3 Agricultural Innovation and Management Division, Faculty of Natural Resources, Prince of Songkla University, Hat Yai 90110, Songkhla, Thailand; saowapa.d@psu.ac.th; 4 Oil Palm Agronomical Research Center, Faculty of Natural Resources, Prince of Songkla University, Hat Yai 90110, Songkhla, Thailand; 5 Hermiston Agricultural Research and Extension Center, Oregon State University, Hermiston, OR 97838, USA; hussaita@oregonstate.edu; 6 Faculty of Technology and Environment, Prince of Songkla University, Phuket Campus, Kathu 83120, Phuket, Thailand

**Keywords:** imagery, spectroscopy, nitrogen, precision, smart farming

## Abstract

Hyperspectral imaging (HSI) is a promising tool in chlorophyll quantification, providing a non-invasive method to collect important information for effective crop management. HSI contributes to food security solutions by optimising crop yields. In this study, we presented a custom HSI system specifically designed to provide a quantitative analysis of leaf chlorophyll content (LCC). To ensure precise estimation, significant wavelengths were identified using optimal-band analysis. Our research was centred on two sets of 120 leaf samples sourced from Thailand’s unique Chaew Khing rice variant. The samples were subjected to (i) an analytical LCC assessment and (ii) HSI imaging for spectral reflectance data capture. A linear regression comparison of these datasets revealed that the green (575 ± 2 nm) and near-infrared (788 ± 2 nm) bands were the most outstanding performers. Notably, the green normalised difference vegetation index (GNDVI) was the most reliable during cross-validation ($R^{2}=0.78$ and RMSE = 2.4 µg∙cm^−2^), outperforming other examined vegetable indices (VIs), such as the simple ratio (RED/GREEN) and the chlorophyll index. The potential development of a streamlined sensor dependent only on these two wavelengths is a significant outcome of identifying these two optimal bands. This innovation can be seamlessly integrated into farming landscapes or attached to UAVs, allowing real-time monitoring and rapid, targeted N management interventions.

## 1. Introduction

Rapid and accurate chlorophyll measurement is essential for real-time crop growth monitoring and nitrogen (N) management in precision agriculture fields [1,2]. Deviations from reasonable chlorophyll levels can help to indicate nutrient deficiencies and diseases [3,4,5]—allowing farmers to address nutrient management, irrigation practices, and disease controls. This proactive approach enhances crop yield and strengthens global food security. Chlorophyll monitoring can assist researchers in interpreting the complex relationship between the physiological and environmental factors that determine plant health, growth status, and agricultural productivity [6,7,8]. Plant genotypes exhibit unique spectral signatures, or patterns of reflected electromagnetic radiation, that can be identified using hyperspectral imaging (HSI) technology [9,10]. The precise estimation of biophysical parameters and accurate monitoring throughout different stages of growth are essential for customising management strategies of crop fields [11].

Achieving accurate prediction of chlorophyll content requires a comprehensive understanding of the distinctive spectral signatures displayed by various crop cultivars that can be achieved using HSI technology. This study demonstrates the application of a custom HSI system for quantifying leaf chlorophyll content (LCC) using the leaf samples of Chaew Khing rice, a rice cultivar indigenous in southern Thailand. By employing optimal-band analysis, this study contributes to the development of cost-effective and efficient technology for agriculture, promoting its broader adoption in the industry.

Plant pigment analysis has conventionally relied on time-consuming and destructive methods such as spectrophotometry [12,13,14], chromatography [15,16], and atomic absorption spectroscopy [17]. These methods require the destruction of leaves and subsequent laboratory analysis, making field assessments impossible in real-time. In contrast, portable chlorophyll metres enable in-field measurements, providing non-invasive analytical capabilities. However, their limited usability is due to the high cost and the ability to only measure small leaf areas that may not accurately represent the chlorophyll content of the entire leaf—making them unsuitable for large-scale imaging. For instance, the commercially available SPAD-502 metre uses a single parameter (the green colour intensity index based on light absorption at 650 nm) to correlate the relative LCC. Despite its fast response time of two seconds, the metre can only measure a small area of 12.57 mm^2^ at a time [18].

HSI is a robust, non-destructive tool with varied applications, especially in mapping the distribution of plant physiological features [19,20,21]. Being an imaging technique, HSI offers the advantage of capturing a large area, such as an entire leaf, in a single measurement. The HSI data is visualised in a three-dimensional map: the first two dimensions capture spatial details, while the third captures spectral attributes. This facilitates data merging from the imaging and conventional spectroscopy domains [22].

HSI integrates image processing with spectral data from remote sensing [23,24,25]. Using a broad range of wavelengths to record an object’s reflectance or emission spectra, HSI can produce a high-dimensional representation by capturing a sequence of monochromatic images at a continuous range of up to thousands of wavelengths [26]. Hyperspectral sensors can collect over one hundred continuous spectral bands with a narrow bandwidth between 500 nm and 2500 nm in the visible to mid-infrared regions of the electromagnetic spectrum. This allows them to provide comprehensive and insightful data regarding chemical composition [27], structural properties, and other characteristics.

With applications ranging from crop analysis to real-time plant health and yield monitoring, HSI has proven invaluable in the agriculture field [5,10,23,28,29,30,31]. Ground-based HSI systems are appropriate for smaller-scale investigations, while airborne setups are suitable for surveying large areas of land. A prominent feature of HSI is its ability to produce complex plant pigment maps, supporting chlorophyll estimation as evidenced by several studies [1,32,33,34,35].

Commercial HSI systems are versatile and can accommodate various applications, but can be costly. However, due to imaging, instrumentation, and optics limitations, developing custom-made HSI systems is challenging [36]. More research and standardisation are necessary to overcome these challenges and optimise the performance of specially designed HSI systems.

Using empirical vegetation indices (VIs) is an efficient and convenient method for estimating plant characteristics, such as their structure and biochemistry, including the chlorophyll content of plants [37]. VIs can be regarded as a modified representation of spectral bands, incorporating established correlations between spectral response and biophysical characteristics. For this reason, they are well-suited for monitoring temporal changes throughout the phenological growth phases [38]. Most VIs are ratios or linear combinations of spectral reflectance in two or more wavelength bands [39] that can be obtained from HSI [37,40]. Consisting of a straightforward transformation of spectral bands, VIs are estimated without bias or presumptions about land cover class, soil type, or climate conditions—enabling the observations of seasonal and long-term changes in a vegetation’s structural, phenological, and biophysical characteristics [41,42,43]. VIs have proven to be a valuable tool for monitoring plant health and productivity, and they are widely applied in fields such as agriculture, forestry, and ecology.

This study evaluated two optical bands that are particularly useful in assessing LCC. Our analysis focused on a linear regression between LCC values obtained from conventional analytical measurements and VIs extracted with HSI. Although our main focus was the Thai Chaew Khing rice cultivar, this study’s flexible approaches and strategies demonstrate broad applicability. They can be modified for other kinds of crops.

The results of this study, including the tested VIs and the model validation, are elaborated upon in a detailed discussion. The optimal-band analysis identified two specific wavelengths as being essential for LCC evaluation. These insights suggest that a streamlined device could be engineered with sensors at these wavelengths and be deployed on UAVs or the ground. This advancement could be instrumental in N management strategies, ensuring precise interventions for agricultural products [44].

## 2. Materials and Methods

To induce variation in chlorophyll content among the collected leaf samples, the fertiliser application was varied by adding different concentrations of urea to the soil across different plant plots. Specifically, urea was added to 12 kg of soil at the following concentrations: 0 g, 3 g, 6 g, 9 g, and 12 g. Rice plants were cultivated in a greenhouse until the tillering stage, when four leaves from each plant plot were randomly pruned at various heights; this was to ensure variation in chlorophyll content among the collected leaves. Each individual leaf was subsequently divided into six equal parts. This produced 120 unique samples in total, each representing a distinct combination of urea content and leaf height. By employing this methodology, we hoped to generate a dataset that covered a broad spectrum of chlorophyll concentrations, enabling us to use our specialised HSI technology for comprehensive chlorophyll quantification.

**Figure 1:**
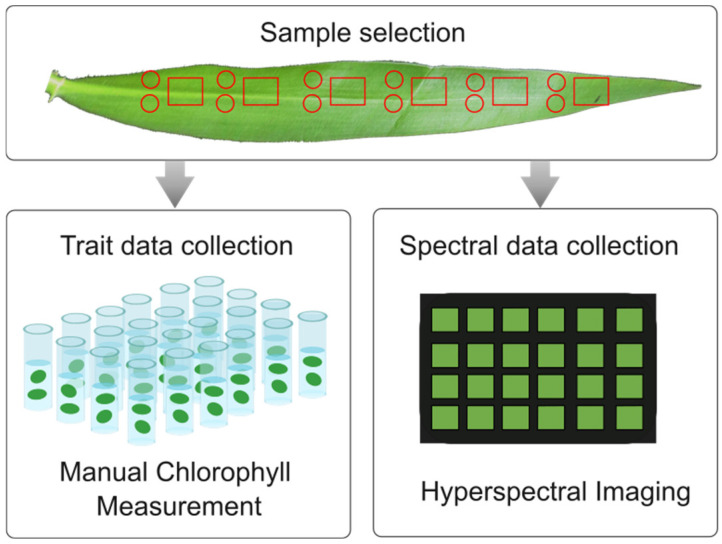
The workflow diagram outlines two sets of leaf samples that were prepared for analytical chlorophyll measurement and hyperspectral imaging to collect the trait and spectral data.

Figure 1 illustrates the sample collection process, involving the division of each leaf into six perpendicular parts along its midrib, resulting in a total of 120 samples. For analytical chlorophyll measurement, two circular discs with an approximate area of 0.84 cm^2^ each were sampled. In addition, a rectangular section of approximately 1.2 cm × 1.5 cm was sampled for HSI to obtain spectral reflectance.

### 2.1. Hyperspectral Imaging

This paper introduces the design of a custom-made laboratory HSI system tailored explicitly for chlorophyll quantification in rice leaves. The system’s design was meticulously refined to eliminate unnecessary complexities and redundant features, resulting in a streamlined, cost-effective, and adaptable configuration that utilises commonly accessible optical components.

The system was specifically engineered to achieve maximum efficiency for the spectral range from 450 nm to 800 nm. This range was chosen to coincide with the photosynthetically active radiation band, which is recognised to be between 400 nm and 700 nm [45]. Additionally, the system was designed to include the critical band in the range of 700 nm to 760 nm, associated with important pigments found in rice leaves (chlorophyll a, chlorophyll b, total chlorophyll, and carotenoids) [33].

To enhance its practicality, the objective was to achieve a spectral resolution of approximately 3 nm while also prioritising an image spatial resolution of 0.5 mm or finer. This facilitated the clear visualisation of chlorophyll distribution across the leaf surface. The imaging spectrometer plays a critical role in HSI systems. The dispersive element used in our custom system is a grism, a transmission grating combined with a prism. This configuration offers several advantages, including a linear spectrometer design that simplifies optical alignment and housing assembly. The grism-based spectrometer design also minimises the field curvature aberration, confining the dispersed rays close to the optical axis [46].

**Figure 2:**
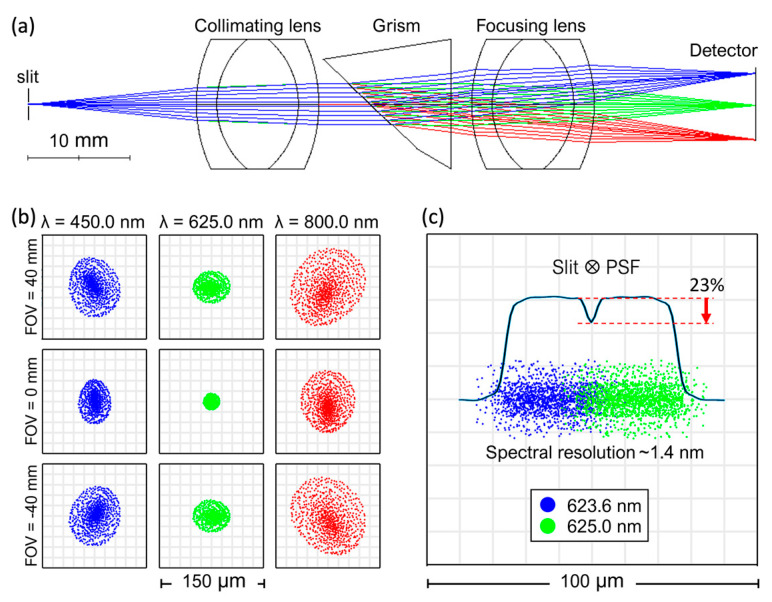
(**a**) The ray tracing simulation for the optical design of the custom hyperspectral imaging system using Zemax OpticStudio at 450 nm, 625 nm, and 800 nm. (**b**) The simulation indicates slight aberration or image distortion across the field of view (FOV) from −40 mm to 40 mm, with resolutions ranging from 1.4 nm to 2.0 nm. At 625.0 nm the best resolution is obtained, while 800.0 nm has the worst resolution. (**c**) Estimation of spectral resolution at the central wavelength near 625.0 nm, based on the convolution of the point spread function and a slit with a width of 25 µm.

In this study, the optical design (based on PF-0035-ALPY 600 purchased from Shelyak Instruments, Le Versoud, France) was achieved via the ray tracing simulation in Zemax OpticStudio (version 23.2.1) with incorporated wavelengths of 450 nm, 625 nm, and 800 nm, as shown in Figure 2a. The figure illustrates the light rays converged using a paraxial lens onto a slit with 25-µm width and 3-mm height. The dispersed light is projected onto the grism-based imaging spectrometer comprising an achromatic triplet collimating lens with a focal length of 20 mm, a grating with a groove density of 600 lines ⋅ mm^−1^, a single prism with a wedge angle of 46°, and an achromatic triplet focusing lens with a focal length of 20 mm.

Figure 2b displays the simulation result of the spot where the field of view (FOV) in the cross-track scan is set to be −40 mm, 0 mm, and 40 mm. All simulated wavelengths demonstrate slight aberration or image distortion across the operational FOV, with resolutions ranging from 1.4 nm to 2.0 nm; 625.0 nm exhibits the best resolution, while 800.0 nm has the worst resolution.

Figure 2c depicts the optimal spectral resolution obtained from the central wavelength of the optical axis (approximately 625.0 nm), evaluated from the convolution of the extracted point spread function (PSF) with a 25-nm slit width. The resolving ability of the two adjacent slit images at 623.6 nm and 625.0 nm in a detector’s plane was assessed using the Rayleigh criterion of minimum resolvable peaks [47].

**Figure 3:**
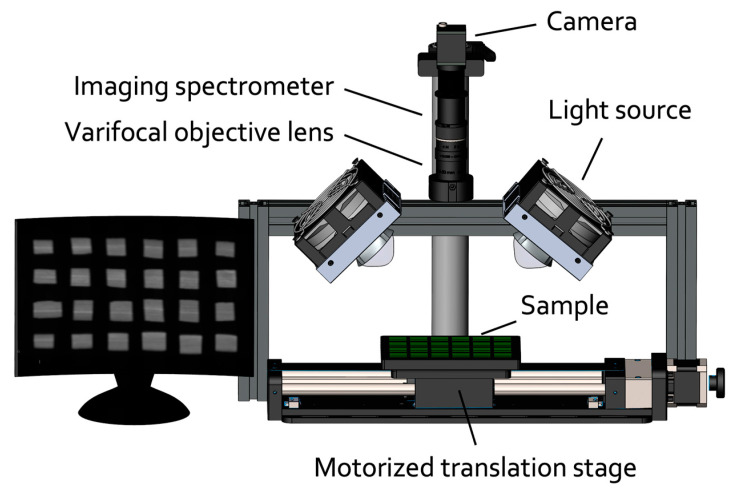
A complete system of custom hyperspectral imaging (HSI).

Figure 3 illustrates the HSI components, including an imaging spectrometer, a varifocal objective lens (Witrue, Shenzhen, China), a translation stage, a broadband light source, and a computing unit. The system’s light source (400 nm to 900 nm) is two 50-W spectrum-extended LED lamps (Shenzhen Learned Optoelectronics Technology, Shenzhen, China). The light is directed onto a specimen with 45° angles of incident. The reflected light is focused onto an order-sorting filter (Thorlabs, NJ, USA) with a cut-on wavelength of 450 nm that helps to prevent an overlap of diffraction orders of adjacent bands. The light is then passed through the objective lens, placed at an optimal working distance (250 mm away from the specimen), and the imaging spectrometer, before being captured by a detector—a 1-inch monochrome CMOS camera (with a resolution of 5488 pixels × 3672 pixels, with each pixel measuring 2.4 µm in size).

The captured image was calibrated using a standard Hg-Ar lamp with a Lorentzian-fitted atomic emission line at 576.96 nm, revealing a 0.72 ± 0.03 nm resolution. This resolution exceeded the initial simulation results (1.4 nm to 2.0 nm). Such resolution allows the HSI system to capture 486 spectral bands, making it suitable for various applications requiring detailed spectral analysis.

**Figure 4:**
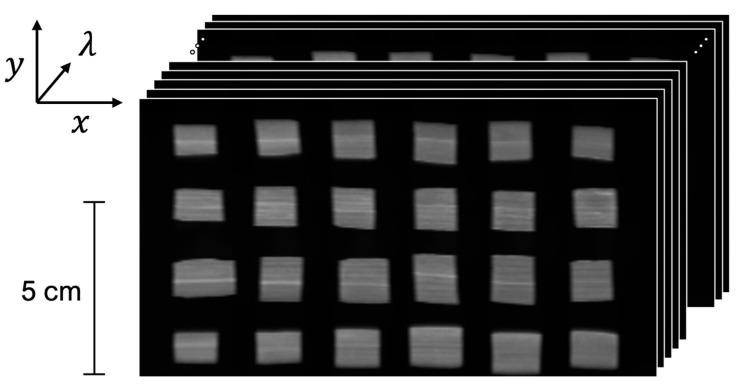
A typical data cube obtained from the custom HSI system.

A linear translational stage (OpticFocus, Singapore) with an increment step of 0.5 mm was used to accomplish the spatiospectral scanning of the leaf reflectance data. Rice leaves were divided into 120 pieces, each measuring 1.2 cm × 1.5 cm, and securely affixed onto five acrylic trays using double-sided tape, ensuring that the leaf surfaces were facing upward; this is the adaxial surface. These sample trays, each containing 24 samples, were then placed on the sample stage for scanning, with the field of view measuring 80 mm (cross-track) × 150 mm (in-track). The images acquired during the scanning process were compiled into a data cube with a spectral dimension consisting of 351 wavelengths and spatial dimensions of 152 pixels (height) × 280 pixels (width), as shown in Figure 4.

### 2.2. Analytical Chlorophyll Measurement

The samples were placed into a glass tube filled with 4 mL of DMF (N,N-dimethylformamide) before being covered and stored in the dark for 24 h at 4 °C. Then, the solution was measured for absorbance at 647 nm (*A*_647_) and 664 nm (*A*_664_) via a spectrophotometer UV-1900i (Shimadzu, Kyoto, Japan). Finally, the leaf chlorophyll content, that is, the total chlorophyll content per unit leaf area in (µg ⋅ cm^−2^), was obtained via

(1) $$LCC=\left(20.27A_{647}+7.04A_{664}\right)\times \frac{4\;mL}{2\times 0.82\;{cm}^{2}},$$

where the term in the parentheses was retrieved from [13]. This measurement determined the total chlorophyll, which includes chlorophyll *a* and chlorophyll *b*. Chlorophyll *a* primarily absorbs red and orange light, while chlorophyll *b* absorbs blue and purple light [26]. The presence of both pigments is crucial for the photosynthetic efficiency of plants [48], as they complement each other’s light absorption spectra.

### 2.3. Optimal-Band Analysis

Linear regression analysis was conducted to optimise the utilisation of LCC information derived from hyperspectral reflectance data. The study involved assigning VIs as independent variables and LCC as the dependent variable. Based on existing literature, three distinct groups of VIs were categorised: the simple ratio (SR), the normalised difference (ND), and the chlorophyll index (CI) [49,50]. The VIs, formulated as combinations of two specific bands (*λ_i_* and *λ_j_*), were expressed via the following equations:

(2) $$SR=\frac{R_{\lambda_{i}}}{R_{\lambda_{j}}}$$

(3) $$ND=\frac{R_{\lambda_{i}}-R_{\lambda_{j}}}{R_{\lambda_{i}}+R_{\lambda_{j}}}$$

(4) $$CI=\frac{R_{\lambda_{i}}}{R_{\lambda_{j}}}-1.$$

**Figure 5:**
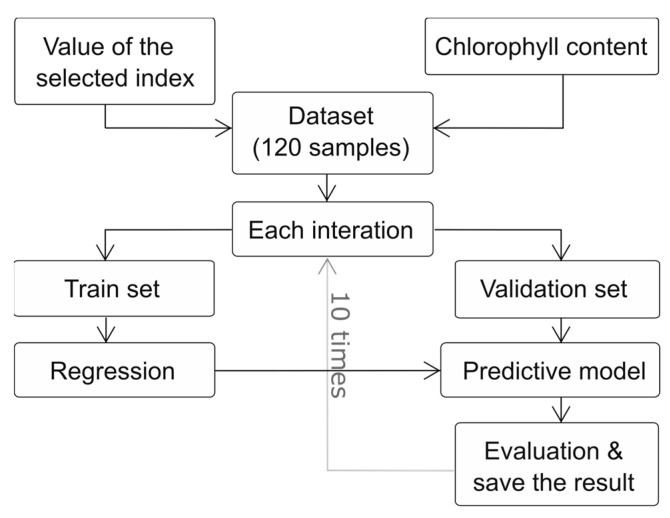
Flowchart of the regression model showing the ten-fold cross-validation process used to train and validate the model. The original dataset containing 120 leaf samples was divided into ten equally sized parts. One part was used as the validation set, and the remaining four parts were used for training. This process was repeated ten times until each part was used as validation.

The analysis focused on two bands within the 450 nm to 800 nm wavelength range. These VIs, formed by combining the selected bands, were used as variables in the regression models. The model flowchart, presented in Figure 5, outlines the process, which incorporated ten-fold cross-validation. This involved equally dividing the original dataset collected from the leaf samples into ten parts, reserving one part as validation data, and using the remaining nine parts for training. The process was iterated ten times to ensure that each part was used for validation.

### 2.4. Statistical Analysis

The model performance was evaluated by taking the mean accuracy of the k-models validation data, using the coefficient of determination:

(5) $$R^{2}=1-\frac{\sum_{i=1}^{N}{\left(y_{i}-{\hat{y}}_{i}\right)}^{2}}{\sum_{i=1}^{N}{\left(y_{i}-{\bar{y}}_{i}\right)}^{2}};$$

and the root mean square error:

(6) $$RMSE=\sqrt{\sum_{i=1}^{N}{\left(\frac{y_{i}-{\hat{y}}_{i}}{y_{i}}\right)}^{2}},$$

where $y_{i}$ and ${\bar{y}}_{i}$ are the measured LCC and its mean, and ${\hat{y}}_{i}$ is the predicted LCC. The evaluation of the model’s performance involved a comparison of the discrepancies between the coefficient of $R^{2}$ and RMSE. A combination of a high $R^{2}$ and a low RMSE suggests reasonable estimate precision and accuracy of the model for predicting the LCC of Chaew Khing rice.

The strength of the model was evaluated using the correlation coefficient, which can be expressed as

(7) $$r=\frac{\sum \left(x_{i}-\bar{x}\right)\left(y_{i}-\bar{y}\right)}{\sqrt{\sum {\left(x_{i}-\bar{x}\right)}^{2}\sum {\left(y_{i}-\bar{y}\right)}^{2}}},$$

where $\bar{x}$ denotes the mean values of the variables. The *r* value lies in the interval [−1, 1], with the negative and positive values indicating negative and positive linear relationships. The strength of the correlation can be interpreted using the absolute value of *r*: negligible (0.00–0.10), weak (0.10–0.39), moderate (0.40–0.69), strong (0.70–0.89), and very strong (0.90–1.00) [51]. It is common to report *r* with the *p*-value. A low *p*-value (typically ≤0.05) suggests the statistical significance of the model, with strong evidence against the null hypothesis.

For each tested VI, a contour map of $R^{2}$ was plotted with wavelengths *λ_i_* and *λ_j_* on the horizontal and vertical axis, respectively. The optimal band was chosen based on the wavelengths that resulted in the highest $R^{2}$. The arithmetic mean position of all points on the contour with the highest $R^{2}$ was calculated to determine its centroid, that was calculated via:

(8) $$\left\{C_{x},C_{y}\right\}=\left\{\frac{M_{10}}{M_{00}},\frac{M_{01}}{M_{00}}\right\},$$

where *M*_10_ and *M*_01_ are the first moments in the x and y directions, respectively. *M*_00_ is the zeroth moment, which is the total area under the contour curve with the highest $R^{2}$.

## 3. Results

### 3.1. Leaf Reflectance

**Figure 6:**
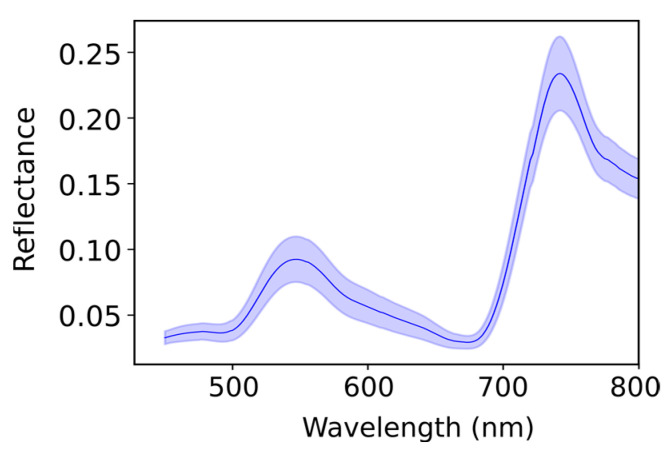
The mean spectral reflectance and standard deviation of 120 leaf samples demonstrate characteristic features of healthy vegetation, including a peak in reflectance within the green light range (500 nm to 600 nm) in the visible region, as well as a distinct increase in reflectance around 700 nm, from the red to the near-infrared regions. There are significant variations in the standard deviation within the green and near-infrared regions.

The mean spectral reflectance curve of the leaf samples is presented in Figure 6. The curve exhibits a peak within the green light region, with lower reflectance observed in the red and blue regions. Additionally, the near-infrared region shows higher reflectance compared to the visible region. 

### 3.2. The Leaf Chlorophyll Content (LCC)

**Figure 7:**
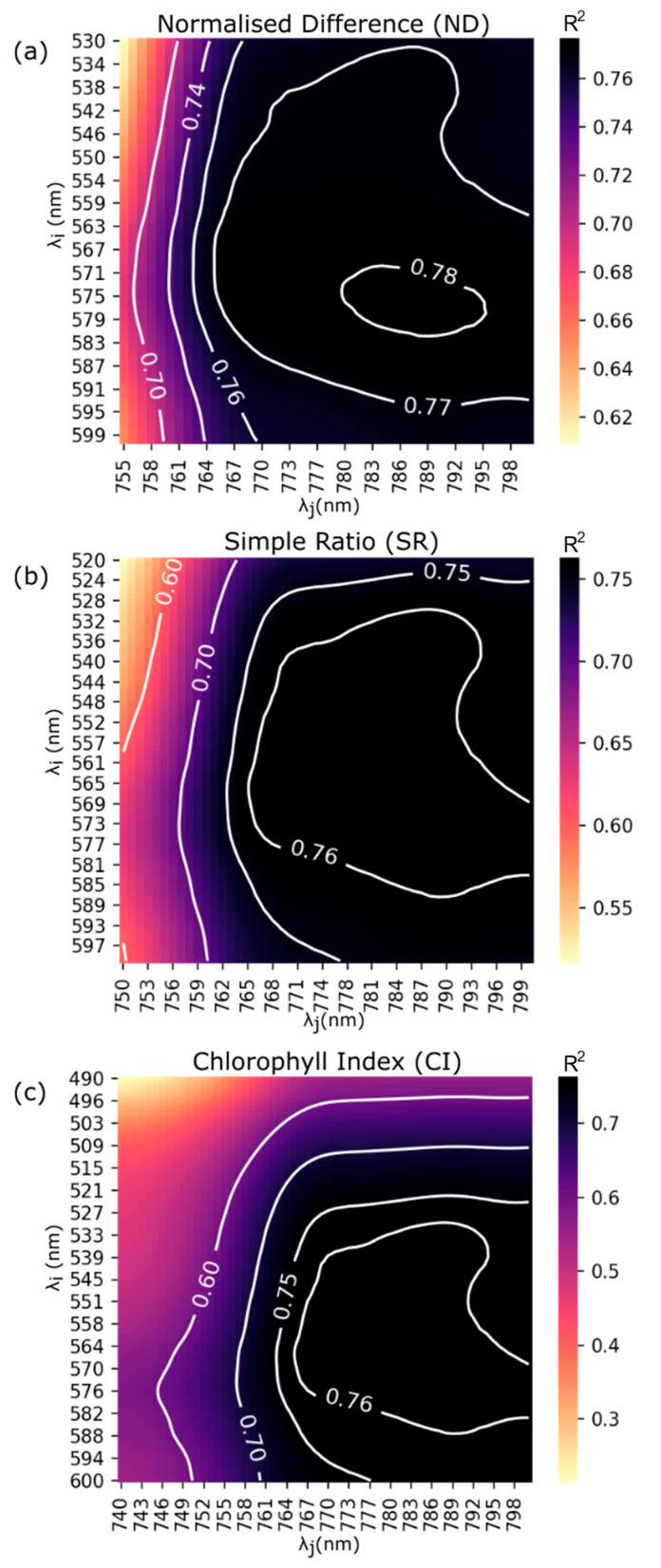
Contour maps of (**a**) the normalised difference (ND), (**b**) simple ratio (SR), and (**c**) chlorophyll index (CI) developed through linear regression analysis, with coefficients of determination (*R*^2^) indicated by the contour lines.

The results of analytical chlorophyll measurement of all samples showed that the obtained LCC range was 4 µg ∙ cm^−2^ to 23 µg ∙ cm^−2^, with a standard deviation of 5 µg∙cm^−2^. To examine the relationship between LCC and the selected VIs, linear regression models were developed and plotted as contour maps in Figure 7, with the $R^{2}$ values indicated by the contour lines.

**Figure 8:**
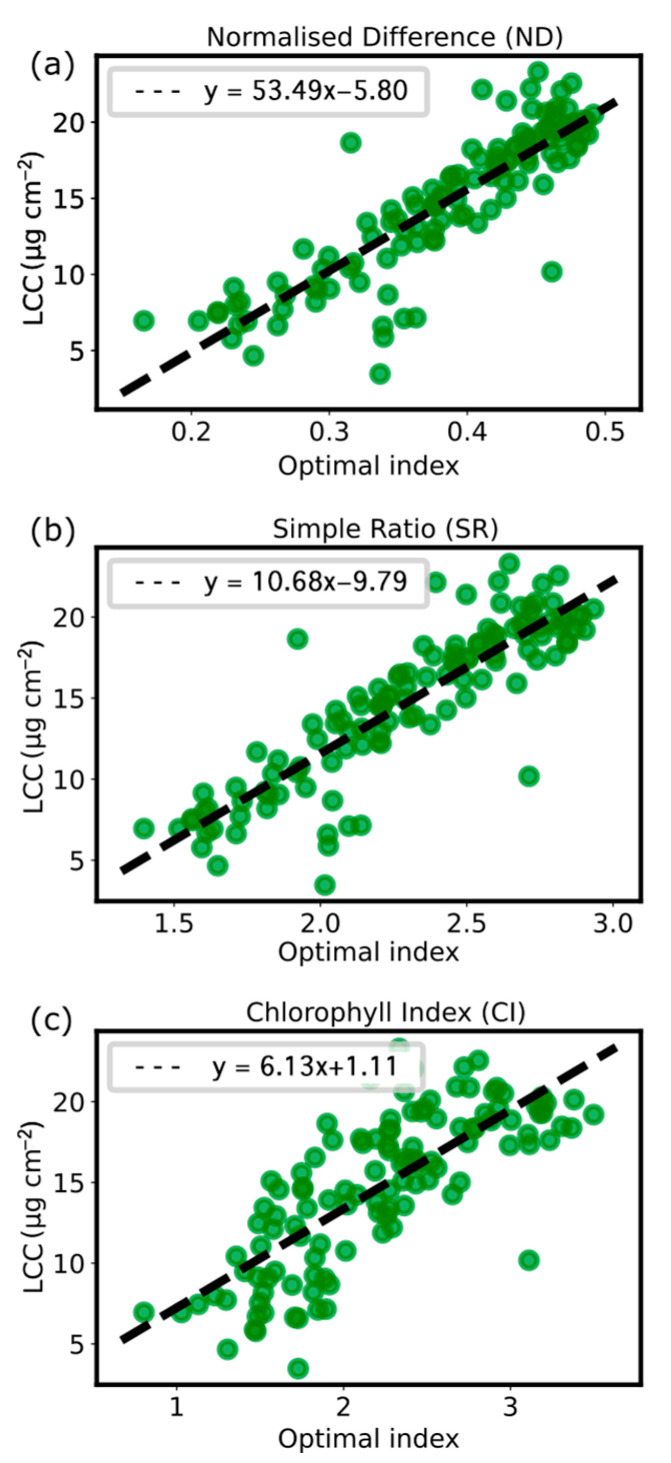
Linear relationships between Leaf Chlorophyll Content (LCC) and the selected VIs: (**a**) normalised difference (ND), (**b**) simple ratio (SR), and (**c**) chlorophyll index (CI). The plot is based on the optimal wavelengths determined for each VI, presenting its stimulated equation.

Linear regression analysis (Figure 8) revealed that all selected VIs were significantly correlated with LCC, albeit with varying degrees of correlation. The optimal wavelengths for each VI were determined by the centroid of the contour map with the highest *R*^2^ value. The corresponding statistics are presented in Table 1. Notably, the optimal wavelengths for all VIs were found to be in the green (572 nm to 575 nm) and near-infrared (784 nm to 788 nm) regions of the electromagnetic spectrum, with a RMSE of 2.4 µg∙cm^−2^ to 2.5 µg∙cm^−2^.

Further analysis of the correlation between LCC and each VI indicated that ND had the strongest correlation with LCC, with an $R^{2}$ value of 0.78, followed by SR and CI, which exhibited comparable performance with $R^{2}$ values of 0.76 each. Regarding the *r*-value, all VIs strongly correlated with LCC, exceeding the significant threshold of 10%. ND and SR exhibited equally high positive correlations with LCC, both with a value of 0.87. In contrast, CI exhibited a slightly weaker positive correlation with LCC, with a coefficient of 0.76.

The statistics above indicate that the VI with the most outstanding performance is the normalised difference between the green and near-infrared regions of the electromagnetic spectrum. This VI, commonly referred to as the green normalised difference vegetation index (GNDVI), is formulated as follows:

(9) $$GNDVI=\frac{R_{\lambda_{788}}-R_{\lambda_{575}}}{R_{\lambda_{788}}+R_{\lambda_{575}}}.$$

The stimulated equation obtained from this study is

(10) $$LCC=\left(53.5\times GNDVI\right)-5.8.$$

**Figure 9:**
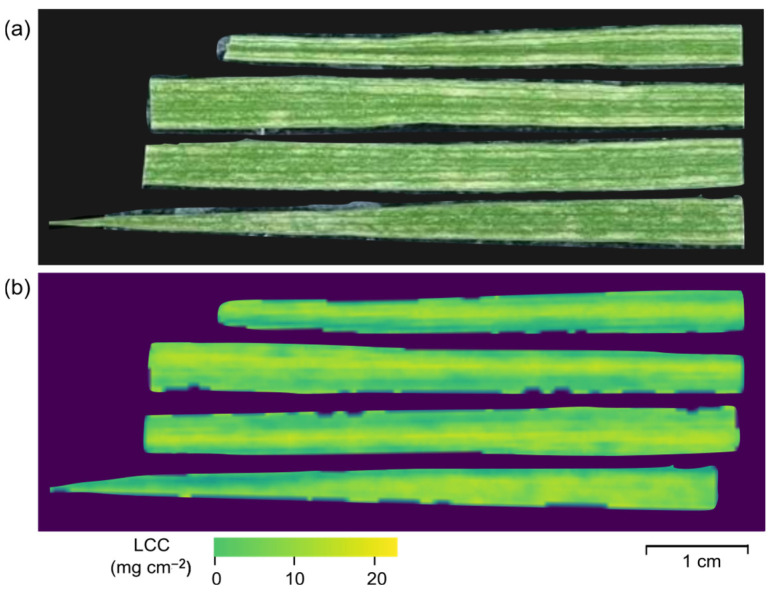
Images of a Chaew Khing rice leaf captured using (**a**) traditional optical (RGB) and (**b**) the green normalised difference vegetation index (GNDVI) calculations. The custom hyperspectral imaging system enables the visualisation of chlorophyll concentration variations.

GNDVI resembles the typical normalised difference vegetation index (NDVI), albeit with the substitution of the green spectral band for the red. GNDVI facilitates the assessment of the photosynthetic activity and exhibits greater sensitivity towards variations in chlorophyll concentration compared to NDVI [52]. GNDVI provides advantages in scenarios where hyperspectral data are deficient in an extreme red channel [53]. VIs using the green wavelength can detect changes in chlorophyll contents at the leaf and canopy scale, and are appropriate for determining the developmental phases and stress levels of a plant [54]. Figure 9 illustrates a Chaew Khing rice leaf, visualised using conventional optical (RGB) images and the LCC quantified via GNDVI. The custom HSI system facilitated the visualisation of chlorophyll concentration differences, further highlighting the potential of this technology in precision agriculture and crop management.

## 4. Discussion

This research demonstrated the utilisation of a custom HSI as a non-invasive tool to evaluate LCC in Cheaw Khing rice. The chlorophyll concentration, the primary photosynthetic pigment responsible for absorbing photosynthetically active radiation in plant leaves, exhibits variation across diverse phenotypes. Nonetheless, the existing body of literature concerning the correlation between alterations in phenotype and LCC in Thai rice still needs to be expanded. Spectral data for the rice samples were collected using a custom HSI with a spectral range spanning from 450 nm to 800 nm. The observed LCC is in the range of 23 µg ⋅ cm^−2^ to 40 µg ⋅ cm^−2^.

A consistent trend in spectral reflectance was observed across all samples, with a decrease in the red and blue regions and an increase in the green and near-infrared regions. Figure 6 displays considerable variations in LCC in the green and near-infrared regions. The visible spectrum (400 nm to 700 nm) embodies the photosynthetically active region of the electromagnetic spectrum. The obtained spectra display typical features of healthy green vegetation, which highly reflects green light (500 nm to 600 nm) but has low reflectance in the blue (450 nm to 500 nm) and the red (600 nm to 700 nm) regions due to the absorption of chlorophyll for photosynthesis [55]. A rapid increase in reflectance around 700 nm, from the red to the near-infrared region—referred to as the ‘red edge’—often appears as a steep slope [56], owing to light scattering in the inter-cellular volume of the leaves mesophyll [57]. This research involved an assessment of VIs formulated as combinations of two wavelengths within the observed hyperspectral regions of 450 nm to 800 nm, namely simple ratio (SR), normalised difference (ND) and chlorophyll index (CI). Linear regression analyses were performed to identify the active bands associated with LCC in Chaew Khing rice for the selected VIs. The results imply that LCC is highly influenced by the spectral bands in the green and near-infrared ranges.

Among the various combinations of wavelength ranges systematically tested, the combination of two bands at the green and near-infrared regions demonstrated the highest degree of accuracy in predicting LCC across all Vis. Our study suggests that the most reliable approach for predicting LCC involves utilising GNDVI, the normalised difference between 575 nm and 788 nm. This VI has been used in numerous studies, proving to be a versatile indicator for predicting not only chlorophyll content [58,59,60,61] but also grain protein content [62], nitrogen concentration [63], and fertilisation rate [64]. The combination of the green and near-infrared spectral bands via GNDVI has demonstrated the potential to enhance the precision of LCC estimation. Furthermore, the proposed methodology presents a novel concept comprising solely two spectral parameters that could yield a more precise estimate of chlorophyll. This implies that implementing hardware in the field would only require light sensors in two bands. Therefore, a viable approach is utilising a hyperspectral system for laboratory analysis and a more cost-effective and compact multispectral system for in-field monitoring.

The versatility of our HSI system can conform to diverse optical configurations, which is a significant benefit for agricultural applications. Modifying the spatial resolution, field-of-view, and working distance of the system can be achieved by altering the lens modules and objective lens focal length, catering to various samples’ diverse requirements. In addition, the system can be equipped with alternative light sources, thereby facilitating the investigation of additional spectroscopic techniques, such as fluorescence imaging and Raman spectroscopy [65]. The adaptability and multifunctionality of our system enable the development of novel imaging techniques and applications, especially for specimens unsupported by conventional methods. The adaptability of our system to diverse optical configurations and supplementary methodologies considerably broadens its potential applications in the domain of agriculture.

Our study has yielded significant insights into the most effective spectral bands for predicting chlorophyll in Chaew rice leaves. This finding holds promise for developing a viable strategy for real-time monitoring of LCC in the field. This study focused on a Thai rice cultivar, which needs spectral data, and the findings contribute to the understanding of locally grown Thai rice. However, this work’s methodology and techniques are versatile compared to other studies [6,8,9,12,34], have broader applicability, and can be extended to other crop varieties. Subsequent investigations should prioritise incorporating more extensive crop samples from diverse growth and planting stages to enhance the classification framework’s reliability and generalisability. Furthermore, to improve the model’s validity, machine learning approaches can be employed to update the model via a larger sample size and incorporate additional factors, such as plant growth stages, planting conditions, plant nitrogen utilization ability, and applied nitrogen fertilization rates, as these factors influenced the nitrogen utilization in Thai rice [66,67,68,69].

The findings of this study have significant implications for developing a compact device capable of LCC measurement using only the identified two wavelengths. This suggests that one can develop a compact, non-destructive device for LCC quantification comprising only two optical sensors at the green and NIR wavelength, offering a cost-effective solution for farmers. Such a device could be integrated into UAVs or deployed in field settings for real-time monitoring of LCC. Leveraging the capabilities of HSI, we identified these informative wavelengths, which opens up possibilities for effective and efficient LCC monitoring in agricultural applications.

## 5. Conclusions

This research demonstrates the use of a custom HSI system (450 nm to 800 nm) to evaluate LCC in Chaew Khing rice. Linear regression analysis revealed that the green and near-infrared spectral bands significantly influenced LCC, especially with their combination as simple ratio and normalised difference, both revealing a correlation coefficient of 0.87. The GNDVI using wavelengths 575 ± 2 nm and 788 ± 2 nm was the most reliable VI for predicting LCC ($R^{2}=0.78$).

The versatility of the HSI system used in this study allows for adjustments in optical configurations, such as spatial resolution, field-of-view, and working distance, catering to diverse sample requirements. Furthermore, the system can be equipped with alternative light sources, enabling the exploration of additional spectroscopic techniques.

While this study focused on a specific Thai rice cultivar, the methodology and techniques employed have broader applicability and can be extended to other crop varieties. Future research should include a broader range of crop samples from different growth stages and planting conditions to enhance the reliability and generalisability of the classification framework. Machine learning approaches can also be considered to update the model with a larger sample size and incorporate additional factors.

The findings of this study have significant implications for developing a compact device that can measure LCC using only the identified two wavelengths. Such a device offers a cost-effective solution for farmers and can be integrated into UAVs or deployed in field settings for real-time monitoring of LCC. Leveraging the capabilities of HSI, the informative wavelengths identified in this research provide opportunities for effective and efficient LCC monitoring in agricultural applications.

## Figures and Tables

**Table 1:** The optimal wavelengths with the corresponding root mean square error (RMSE) and correlation coefficient (r) for the linear regressions models of Leaf Chlorophyll Content (LCC) versus the VIs, namely normalised difference (ND), simple ratio (SR), and chlorophyll indices (CI). (Model significant at the 0.01 level of probability).

VI	Formula	Optimal *λ_i_* nm	Optimal *λ_j_* nm	Determination $\mathrm{Coefficient}\;(R^{2})$	Root Mean Square Error (RMSE) µg∙cm^−2^	Correlation Coefficient (*r*)
ND	$\frac{R_{\lambda_{i}}-R_{\lambda_{j}}}{R_{\lambda_{i}}+R_{\lambda_{j}}}$	788 ± 2	575 ± 2	0.78	2.40	0.87
SR	$\frac{R_{\lambda_{i}}}{R_{\lambda_{j}}}$	786 ± 4	572 ± 4	0.76	2.47	0.87
CI	$\frac{R_{\lambda_{i}}}{R_{\lambda_{j}}}-1$	784 ± 4	574 ± 4	0.76	2.47	0.76

## Data Availability

The data presented in this study are available in this article.
